# Supplementary material for: Hidden long-range memories of growth and cycle speed correlate cell cycles in lineage trees
Source: eLife. 2020 Jan 23;9:e51002. doi: 10.7554/eLife.51002 (PMC7018508; doi:10.7554/eLife.51002)
Supplement: Figure 1—source data 1. — ‘Corrected’ refers to the number of fully observed generations; only these were used, in order to correct for censoring bias. ‘Figures’ refers to main text figures and the respective supplements. [file elife-51002-fig1-data1.pdf]

Experiment ID	Cell line	Perturbation	Obs. time	Total generations	Corrected	Figures
rep1	TET21N	*MYCN*-overexpression	140h	10	6	Fig. 1
rep2			141h	11	7	
rep3			120h	9	5	
-myc1		*MYCN*-inhibition, 1μg/ml doxycycline	166h	9	5	Fig. 4
-myc2		*MYCN*-inhibition, 1μg/ml doxycycline	141h	8	5	
rap1		*mTOR*-inhibition, 40nM rapamycin	117h	8	5	Fig. 4
rap2		*mTOR*-inhibition, 20nM rapamycin	115h	7	5	
esc1	R1 mESCs	non-transformed	>103h	7		Fig. 5
esc2			>101h	10		
esc1			>86h	8		
