# Supplementary material for: Hidden long-range memories of growth and cycle speed correlate cell cycles in lineage trees
Source: eLife. 2020 Jan 23;9:e51002. doi: 10.7554/eLife.51002 (PMC7018508; doi:10.7554/eLife.51002)
Supplement: Figure 3—source data 1. — Cell size was assumed to increase exponentially except for MYCN-inhibited cells, which were modelled by a logistic growth process with a fixed normalised maximum cell size sm⁢a⁢x equal to 20 times the threshold division size. The bottom two rows show the parameters used for simulated perturbations of growth cycle progression, respectively. [file elife-51002-fig3-data1.pdf]

Experiment ID	k	σ_g	a	γ	μ	σ_p
rep1	0.0375	0.02	0.56	0.8	2.7	0.4
rep2	0.045	0.02	0.16	0.6	2.66	0.2
rep3	0.0405	0.02	0.27	0.58	2.76	0.22
-myc1 (logistic)	0.0315	0.04	0.6	0.65	3.14	0.18
-myc2 (logistic)	0.033	0.02	0.3	0.6	3.14	0.22
rap1	0.037	0.05	0.94	0.96	2.66	0.4
rap2	0.0285	0.04	0.82	0.84	2.89	0.44
esc1	0.057	0.05	0.72	0.92	2.26	0.38
esc2	0.0695	0.07	0.89	0.98	2.09	0.41
esc3	0.0685	0.04	0.8	0.8	1.78	0.62
rep1 ($k - 10\%$)	0.03375	0.02	0.56	0.8	2.7	0.4
rep1 ($\mu + 7.5\%$)	0.0375	0.02	0.56	0.8	2.9025	0.4
