# Supplementary material for: Hidden long-range memories of growth and cycle speed correlate cell cycles in lineage trees
Source: eLife. 2020 Jan 23;9:e51002. doi: 10.7554/eLife.51002 (PMC7018508; doi:10.7554/eLife.51002)
Supplement: Supplementary file 1. [file elife-51002-supp1.doc]

| **Key Resources Table** | | | | |
| --- | --- | --- | --- | --- |
| **Reagent type (species) or resource** | **Designation** | **Source or reference** | **Identifiers** | **Additional information** |
| cell line (*Homo-sapiens*) | TET21N | Lutz et al., 1996 | RRID: CVCL_9812 | *MYCN* overexpressing |
| cell line (*Mus musculus*) | R1 mESC2 | Filipczyk et al., 2015 |  | Filipczyk et al. kindly shared their tracked time-lapse data |
| antibody | anti-human N-MYC (Mouse monoclonal) | Santa Cruz Biotechnology | cat#sc-53993; RRID: AB_831602 | FACS (1 µg per sample) |
| antibody | AlexaFluor488  IgG anti-mouse (goat, polyclonal) | LifeTechnologies | cat#A-11001; RRID: AB_  2534069) | secondary flouresence-conjugated antibody, FACS (1 µg per sample) |
| chemical compound, drug | rapamycin | Calbiochem | 553210-100UG | tested doses 20nM, 40nM and 80 nM |
| software, algorithm | Fiji | [http://fiji.sc](http://fiji.sc/) | RRID:SCR_002285 | cell tracking |
| software, algorithm | MATLAB | Mathworks | RRID:SCR_001622 | Tracking data analysis and growth-progression model |
| software, algorithm | R | [R Project for Statistical Computing](https://scicrunch.org/resources/Tools/record/nlx_144509-1/e373879a-1ea4-570a-8494-458ed032636b/search?q=R&l=R) | RRID:SCR_001905 | growth-progression model |
| software, algorithm | NILS! Growth-progression model |  |  | growth-progression model |
| software, algorithm | Python | Python | RRID:SCR_008394 | BAR model |
| other | FxCycle Violet Stain (DNA cycle dye) | Thermo Fischer Scientific | cat# F10347 |  |
| other | deposited RNA-Seq data of TET21N cells | Ryl et al., 2017 | GEO: GSE98274 | analysed the untreated controls |
