# Supplementary material for: Hidden long-range memories of growth and cycle speed correlate cell cycles in lineage trees
Source: eLife. 2020 Jan 23;9:e51002. doi: 10.7554/eLife.51002 (PMC7018508; doi:10.7554/eLife.51002)
Supplement: Transparent reporting form [file elife-51002-transrepform.pdf]

1st Floor
24 Hills Road
Cambridge CB2 1JP, UK

P 01223 855340
W elifesciences.org
T @elife

eLife's transparent reporting form

We encourage authors to provide detailed information *within their submission* to facilitate the interpretation and replication of experiments. Authors can upload supporting documentation to indicate the use of appropriate reporting guidelines for health-related research (see EQUATOR Network), life science research (see the BioSharing Information Resource), or the ARRIVE guidelines for reporting work involving animal research. Where applicable, authors should refer to any relevant reporting standards documents in this form.

If you have any questions, please consult our Journal Policies and/or contact us: editorial@elifesciences.org.

Sample-size estimation

- You should state whether an appropriate sample size was computed when the study was being designed
- You should state the statistical method of sample size computation and any required assumptions
- If no explicit power analysis was used, you should describe how you decided what sample (replicate) size (number) to use

Please outline where this information can be found within the submission (e.g., sections or figure legends), or explain why this information doesn't apply to your submission:

We did not use power analysis to pre-calculate sample size; in particular, no statistical testing of null hypotheses was involved in our work that would warrant predetermined sample sizes. Instead, we measured large data sets on cell lineage trees by live-cell imaging to obtain a sound basis for statistical model selection. Specifically, the extent of data collected was determined by the following considerations:

1. Live-cell imaging of neuroblastoma cells with ectopic MYCN expression under unperturbed culture conditions, MYCN inhibition and rapamycin treatment: Cells were imaged for as long as constant culture conditions could be maintained to guarantee exponential growth by avoiding overcrowding. This was verified by measuring exponential population growth as well as further measures of stationarity of culture conditions, including the distribution of cell-cycle durations of individual cell lineages (Figure 1-Figure supplement 1A-C).
2. For each movie, it was verified that the finite observation duration was sufficient to reliably calculate all correlations between related cells shown (Figure 1-Figure supplement 2A, Appendix 1 Data analysis-censoring).
3. Within each movie, the vast majority of cells was tracked. Cells that left the field of view early during the movie, leading to small family trees, were excluded from further analysis.

eLife Sciences Publications, Ltd is a limited liability non-profit non-stock corporation incorporated in the State of Delaware, USA, with company number 5030732, and is registered in the UK with company number FC030576 and branch number BR015634 at the address 1st Floor, 24 Hills Road, Cambridge CB2 1JP | August 2014

Replicates

- You should report how often each experiment was performed
- You should include a definition of biological versus technical replication
- The data obtained should be provided and sufficient information should be provided to indicate the number of independent biological and/or technical replicates
- If you encountered any outliers, you should describe how these were handled
- Criteria for exclusion/inclusion of data should be clearly stated
- High-throughput sequence data should be uploaded before submission, with a private link for reviewers provided (these are available from both GEO and ArrayExpress)

Please outline where this information can be found within the submission (e.g., sections or figure legends), or explain why this information doesn't apply to your submission:

We performed independent biological replicate experiments as follows:

Cells were cultured by the same protocol. For ectopic MYCN expressing neuroblastoma cells, three replicate experiments were performed, labelled rep1, rep2 and rep3 in the supplements to Figures 1-3. In total 68 cell lineages were tracked and analysed, as illustrated in Figure 1-Figure supplement 1D, up to 10 generations deep.

For the each of the perturbation experiments, with inhibition of MYCN expression and rapamycin treatment, two biological replicates were performed, labelled –myc1 and –myc2 and rap1 and rap2, respectively, as stated in Figure 4-Figure supplement 1.

No data were excluded from the analysis, except for a small number of cells that left the observation window too early, resulting in too short lineage trees to reliably calculate correlations of cell-cycle times (see Point 3 above and stated in the legend to Figure 1-Figure supplement 1).

eLife Sciences Publications, Ltd is a limited liability non-profit non-stock corporation incorporated in the State of Delaware, USA, with company number 5030732, and is registered in the UK with company number FC030576 and branch number BR015634 at the address 1st Floor, 24 Hills Road, Cambridge CB2 1JP | August 2014

Statistical reporting

- Statistical analysis methods should be described and justified
- Raw data should be presented in figures whenever informative to do so (typically when N per group is less than 10)
- For each experiment, you should identify the statistical tests used, exact values of N, definitions of center, methods of multiple test correction, and dispersion and precision measures (e.g., mean, median, SD, SEM, confidence intervals; and, for the major substantive results, a measure of effect size (e.g., Pearson's r, Cohen's d)
- Report exact p-values wherever possible alongside the summary statistics and 95% confidence intervals. These should be reported for all key questions and not only when the p-value is less than 0.05.

Please outline where this information can be found within the submission (e.g., sections or figure legends), or explain why this information doesn't apply to your submission:

Except for a test each on spatial trends and for comparison of mTOR mRNA levels (see below), the work does not involve statistical testing of hypotheses. Instead, Bayesian model selection was used to extract mechanistic information from the experimental data. Summary statistics of the experimental data are given with 95% confidence intervals (obtained via non-parametric bootstrap at the level of lineage trees, see below), allowing the reader to judge measurement error.

Specifically, summary statistics comprising median and interquartile range of cell-cycle length distributions for each replicate experiment are shown and described in the figure legends (Figure 1B, Figure 1-supplement 1B, Figure 4C, Figure 4-Figure supplement 1C, Figure 5A, Figure 5-Figure supplement 1A). Since cycle length is not normally distributed, the median and interquartile range provide a better summary of the distribution than mean and standard deviation.

Spearman rank correlation coefficients of cell-cycle durations were calculated for each experiment; details are given in Figure 1-Figure supplement 2B. Spearman rank correlations were chosen over Pearson correlations due to the non-normal distribution of cycle lengths and to provide robustness to outliers. 95% confidence bounds on the coefficients were determined using bootstrap resampling on the level of entire lineage trees. Resampling on the level of individual pairs of related cells would neglect the correlations between cells of the same tree and thus underestimate variability (Appendix 1 – Data analysis-Correlation coefficients).

mTOR mRNA expression in ectopic MYCN expressing and MYCN-inhibited cells was compared using a paired t-test, assuming normally distributed biological and technical noise, as outlined in the figure legend in Figure 4-Figure supplement 1A.

Movies were tested for confounding effects of temporal drift in cycle lengths over the duration of the experiment by calculating partial correlations with respect to time. This procedure detects potential linear drifts (Figure 1-Figure supplement 2C). In addition, cells were grouped by their birth time and cycle lengths were analysed by moving window averaging to detect potential non-linear drifts (Figure 1C, Figure 1-Figure supplement 1C, Appendix 1-Data analysis-Cell cycle length distribution over time).

Potential confounding effects of spatial correlations between cells were tested by comparing cycle length distributions of groups of cells in spatial proximity with that of the whole population using Wilcoxon rank sum tests, again accounting for the nonparametric nature of the data (Figure 1-Figure supplement 2D). In addition, cousin correlations were probed for spatial trends by testing whether spatially closer cousins displayed a higher correlation (Figure 1-Figure supplement 2E).

eLIFE
1st Floor
24 Hills Road
Cambridge CB2 1JP, UK
P 01223 855340
W elifesciences.org
T @elife

(For large datasets, or papers with a very large number of statistical tests, you may upload a single table file with tests, Ns, etc., with reference to sections in the manuscript.)

Group allocation

- Indicate how samples were allocated into experimental groups (in the case of clinical studies, please specify allocation to treatment method); if randomization was used, please also state if restricted randomization was applied
- Indicate if masking was used during group allocation, data collection and/or data analysis

Please outline where this information can be found within the submission (e.g., sections or figure legends), or explain why this information doesn't apply to your submission:

Group allocation is not applicable to the work.

Additional data files ("source data")

- We encourage you to upload relevant additional data files, such as numerical data that are represented as a graph in a figure, or as a summary table
- Where provided, these should be in the most useful format, and they can be uploaded as "Source data" files linked to a main figure or table
- Include model definition files including the full list of parameters used
- Include code used for data analysis (e.g., R, MatLab)
- Avoid stating that data files are "available upon request"

Please indicate the figures or tables for which source data files have been provided:

Source data on analysed lineage tress included for:
Figure 1, TET21N cells without perturbation
Figure 4, TET21N cells with either stable MYCN knockdown or rapamycin treatment.

The computational code used in the paper is available at https://github.com/hoefer-lab/bar-cycle.

eLife Sciences Publications, Ltd is a limited liability non-profit non-stock corporation incorporated in the State of Delaware, USA, with company number 5030732, and is registered in the UK with company number FC030576 and branch number BR015634 at the address 1st Floor, 24 Hills Road, Cambridge CB2 1JP | August 2014
